# Evolution of MIR159/319 microRNA genes and their post-transcriptional regulatory link to siRNA pathways

**DOI:** 10.1186/1471-2148-11-122

**Published:** 2011-05-12

**Authors:** Yang Li, Chaoqun Li, Guohui Ding, Youxin Jin

**Affiliations:** 1 School of Life Sciences, Shanghai University, 200444, Shanghai, P.R.China; 2 State Key Laboratory of Molecular Biology, Institute of Biochemistry and Cell Biology, Shanghai Institutes for Biological Sciences, Chinese Academy of Sciences, 320 Yue Yang Road, 200031, Shanghai, China; 3 Department of Gastroenterology, Shanghai Public Health Clinical Center Affiliated to Fudan University, 2901 Caolang Road, 201508, Shanghai, China; 4 Bioinformatics Center, Key Lab of Systems Biology, Shanghai Institutes for Biological Sciences, Chinese Academy of Sciences, 200031, Shanghai, China

## Abstract

**Background:**

MicroRNAs (miRNAs) are prevalent and important endogenous gene regulators in eukaryotes. MiR159 and miR319 are highly conserved miRNAs essential for plant development and fertility. Despite high similarity in conservation pattern and mature miRNA sequences, miR159 and miR319 have distinct expression patterns, targets and functions. In addition, both MIR319 and MIR159 precursors produce multiple miRNAs in a phased loop-to-base manner. Thus, MIR159 and MIR319 appear to be related in origin and considerably diverged. However the phylogeny of MIR159 and MIR319 genes and why such unusual style of miRNA production has been conserved during evolution is not well understood.

**Results:**

We reconstructed the phylogeny of MIR159/319 genes and analyzed their mature miRNA expression. The inferred phylogeny suggests that the MIR159/319 genes may have formed at least ten extant early-branching clades through gene duplication and loss. A series of duplications occurred in the common ancestor of seed plants leading to the original split of flowering plant MIR159 and MIR319. The results also indicate that the expression of MIR159/319 is regulated at post-transcriptional level to switch on the expression of alternative miRNAs during development in a highly spatio-temporal specific manner, and to selectively respond to the disruption of defensive siRNA pathways. Such intra-stem-loop regulation appears diverged across the early-branching clades of MIR159/319 genes.

**Conclusions:**

Our results support that the MIR159 and MIR319 genes evolve from a common ancestor, which is likely to be a phased stem-loop small RNA. Through duplication and loss of genes this miRNA gene family formed clades specific to moss, lycopods, gymnosperms and angiosperms including the two major clades of flowering plants containing the founding members of MIR319 and MIR159 genes in *A.thaliana*. Our analyses also suggest that some MIR159/319 have evolved into unusual miRNA genes that are regulated at post-transcriptional level to express multiple mature products with variable proportions under different circumstances. Moreover, our analyses reveal conserved regulatory link of MIR159/319 genes to siRNA pathway through post-transcriptional regulation.

## Background

MicroRNA is a class of approximately 21-nucleotide (nt) small RNA that regulate endogenous gene expression in eukaryotes ranging from single cellular green alga to mammals and flowering plants. Many biological processes including development, senescence, metabolism and stress responses are regulated by miRNAs [1,2]. In plants, miRNAs are distinguished from other small RNAs in that they are excised precisely from specific positions on stem-loop precursors by DICER-LIKE 1 (DCL1) with the assistance of HYL1 and SERRATE [2,3]. The mature miRNAs are 2'-o-methylated at 3' end by HEN1 and exported into cytoplasm. After being loaded into RNA Induced Silencing Complex (RISC) the miRNAs anneal to the complementary sites on target mRNAs to impose translational repression or/and cleavage of target mRNAs [2].

MiR159 and miR319 are highly conserved miRNAs that play important roles in plant growth, morphogenesis and reproduction [4]. In *Arabidopsis*, the 21-nt mature miR159 and miR319 share 17 identical nucleotides. However, they have distinct target genes due to sequence specificity and different expression patterns [5]. MiR159 restricts the expression of some MYB transcription factors, while miR319 targets a subset of TCP transcription factor genes [5-8]. Expression of miR159 is abundant and widespread over the whole plant, while miR319 is expressed at much lower level and confined to specific tissues and developmental stages [9]. MiR159 can not induce mRNA cleavage of the miR319-targeted TCP transcription factors due to sequence specificity. Although the miR319 can also mediate the cleavage of MYB33 and MYB65 mRNAs, miR319 does not normally make significant contribution to the regulation of MYB because of its low and specialized expression [5]. The interplay of miR159 and its target MYB is involved in the regulation of vegetative growth, flowering time, anther development, seed shape and germination [8,10,11]. In contrast to miR159, miR319 and corresponding targets regulate embryonic patterning, jasmonate synthesis, leaf morphogenesis and senescence [6,12,13]. A recent study also showed the regulatory role of miR319 in the development of petal and stamen [14].

A distinguished feature of the MIR159 and MIR319 genes conserved from moss to flowering plant is that their stem-loop precursors usually have elongated stem structure. A loop-proximal segment on the MIR159/319 stem-loop precursor outside of the miRNA and miRNA* is also conserved, albeit to a much weaker extent [6,15,16]. Recent studies show that the MIR159 and MIR319 precursors are processed from loop to base to liberate three phased miRNA duplexes [15,17,18]. The miR319 or miR159 can not be efficiently excised without correct processing of the loop-proximal miRNA duplex [17]. According to this scenario, when miR159 or miR319 duplexes are produced the same amount of miRNAs derived from the other two duplexes must be generated. However, only the mature miR159 or miR319 are abundantly expressed while the alternative miRNAs are expressed at low levels, indicating that only miR159 or miR319 are incorporated into RISC and stabilized. Conservation of such an uncommon pattern in biogenesis during the long time evolution of land plants is inexplicable by the known function of miR159 and miR319. The underlying significance of such unusual style of maturation for the MIR159/319 genes remains unknown.

With similarities in sequence, conservation pattern and biogenesis, miR159 and miR319 might originate from a common ancestor. Conversely, differences in expression patterns, target genes and functions indicate that miR159 and miR319 are evolutionarily distinct groups. Although clear evidences are absent to support the common origin of miR159 and miR319 [5], they are categorized into one miRNA gene family in the miRbase and some other studies [7,19]. Hypothesis has been proposed that the miR159 might evolve from miR319 because miR159 seems more specialized in the spectrum of targets [5]. However, the phylogeny of the two hypothesized subfunctionalized groups remains unknown.

In order to study the evolution of MIR159/319 family in land plants, we collected and analyzed a broad set of MIR159/319 stem-loops from a wide range of plant species. Then, we reconstructed the phylogenetic tree of MIR159/319 from a structural alignment using RNA models for paired and unpaired nucleotides, thus revealing the evolutionary history of this miRNA gene family. Our analysis also suggests that post-transcriptional regulations of MIR159/319 expression have evolved during plant evolution, through which MIR159/319 miRNAs respond to defensive siRNA deficiency. This provides explanations for the conserved three-duplex dicing of the MIR159/319 precursors as a framework for an unknown product-tunable mechanism controlling output from their stem-loops. Taken together, our analysis revealed the evolutionary history of MIR159/319 genes, conservation and variability of post-transcriptional regulation across their extant clades during phylogeny.

## Results

### Homology search for MIR159/319 genes

**Figure 1:**
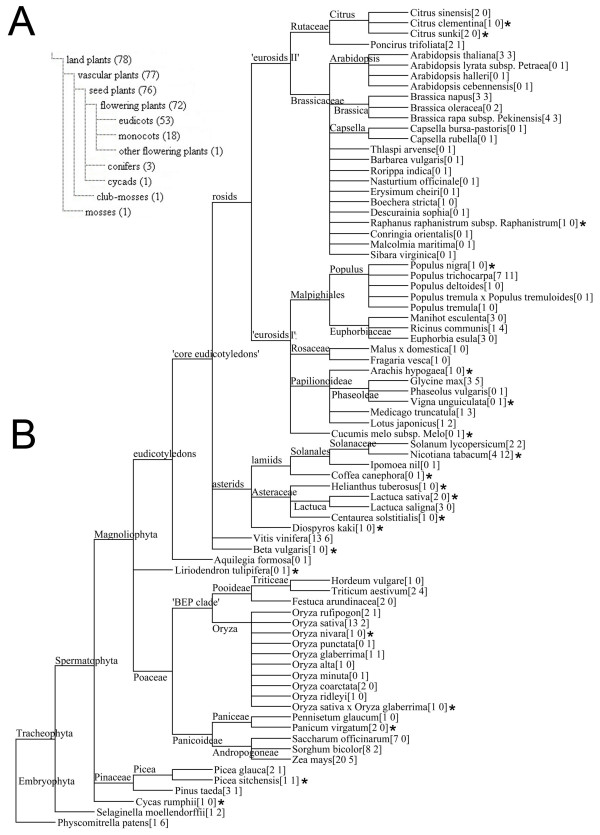
**Taxonomic distribution of the collected MIR159/319 genes**. **A**: The number of species in the major clades of land plants is indicated in brackets, in which at least one MIR159/319 gene was identified. **B**: The number of MIR159/319 genes in land plant species is indicated by two separate numbers in square brackets, the first for MIR159 and the second for MIR319. The asterisks indicate species that harbor MIR159/319 genes newly identified in this study.

In order to get a comprehensive view of the MIR159/319 gene evolution in land plants, we performed homology search like previous studies to collect the MIR159/319 stem-loop sequences from available data resources as exhaustive as possible [7,16,20] (see Methods). Along with the published sequences, we obtained 251 MIR159/319 genes and 27 candidates from 76 land plant species (Figure 1 and Additional File 1). Notice that 3 genes from two species, *Oryza alta *and *Oryza glaberrima*, in Figure 1 are not included and analyzed further because full-length stem-loop sequence could not be retrieved from the database. MIR159/319 genes from most of the major land plant clades are obtained making it possible for an informative phylogenetic reconstruction.

### Classification of MIR159/319 genes according to conservation pattern

**Figure 2:**
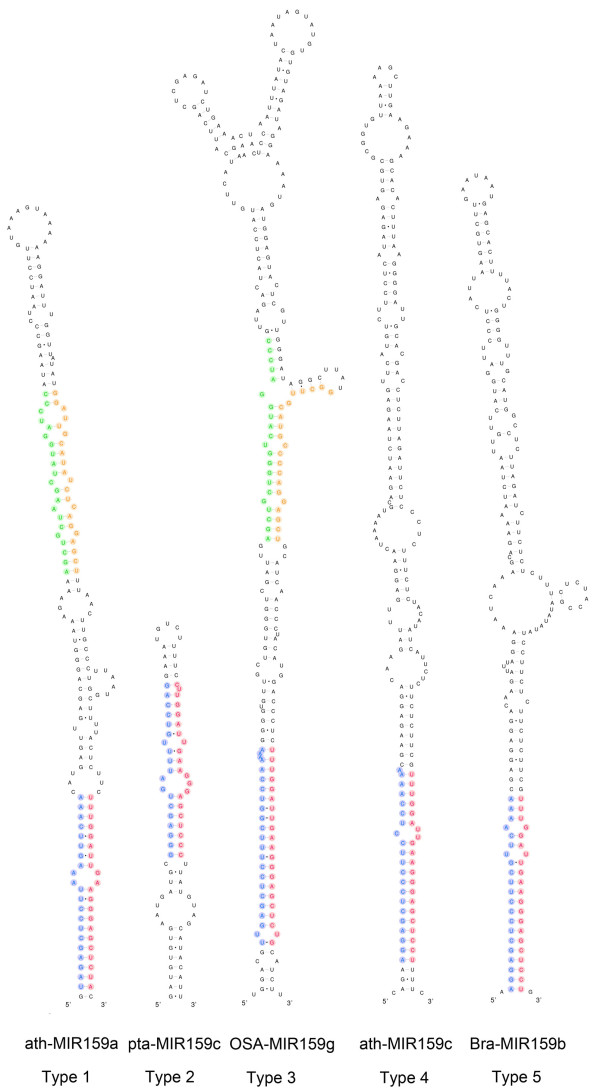
**Examples of the five types of MIR159/319 precursor stem-loops**. The sequences corresponding to the four conserved partitions are highlighted: red, miR; blue, miR*; green, ACR5; orange, ACR3. Notice that the conserved regions may have slight differences from the true miRNA:miRNA* duplexes.

**Figure 3:**
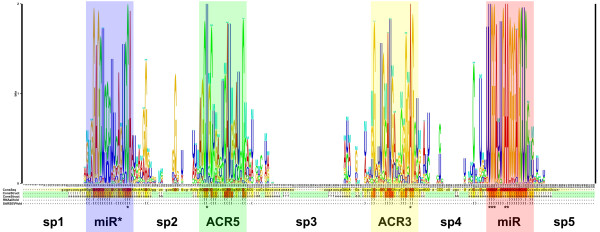
**"Bi-duplex" conservation pattern of type 1 MIR159/319 precursor stem-loops in land plants**. From top to bottom: structure logo, consensus sequence and consensus RNA secondary structures predicted using the software indicated on the left. Consensus nucleotides are marked with an asterisk. The names of the nine partitions are indicated on the bottom. The four well-conserved partitions are highlighted in different colours that designate the boundaries of the partitions.

In the species of *Brassicaceae *closely related to *Arabidopsis thaliana*, the most conserved part of MIR319 genes is the sequence of the precursor stem-loop [21], which is also conserved in distantly related species [6,15,16]. Mutations accumulated in the miRNA stem-loops during evolution may provide useful information for inferring the phylogeny of miRNA families with ancient origin. We aligned the MIR159/319 stem-loops considering both sequence and RNA secondary structure by a semi-automated strategy (see Methods). We found that not all MIR159/319 precursors have conserved elongated stem and can be well-aligned in the loop-proximal regions. According to sequence and structural conservation of the loop-proximal regions, we classified the MIR159/319 genes into 5 types (Table 1 and Figure 2). Type 1 stem-loops show a "bi-duplex" conservation pattern similar to previously discovered [6,15,16]. However we observed much stronger conservation in the loop-proximal regions (Figure 3). Clearly, two duplexes are conserved in both sequence and secondary structure across 71 land plant species from moss to flowering plants (Figure 2, Figure 3 and Table 1). The structural logo shows pronounced similarity in the two loop-proximal regions comparable to that of the miR* region, unlike the poorly conservation of these regions found previously [6,16] (Figure 3 and Additional file 2). This is probably due to the preclusion of other types of MIR159/319, which do not have well-conserved loop-proximal regions. Consensus structure and nucleotides were predicted for the type 1 MIR159/319 genes. Ten consensus base-pairs were detected including a highly conserved U:A pair in the loop proximal regions (Figure 3 and Additional file 2). Noticeably, there is a consensus interior loop in the duplex of miR159/319 and the corresponding miRNA* indicating that selection pressure favor the structural pattern as a whole. Nine partitions can be assigned in the type 1 MIR159/319 stem-loops according to conservation pattern. There are four conserved partitions: miR, miR*, Alternative Conserved Region at 5'arm (ACR5) and 3'arm (ACR3), which accommodate most of the conserved paired or unpaired nucleotides. The conserved partitions are separated by five space partitions (sp1 to sp5) (Figure 3 and Additional file 2). This type is the most widely distributed, while the other types do not have well-conserved loop-proximal duplexes and exist only in a few plant species (Figure 2, Figure 3, Table. 1, and Additional file 1).

**Table 1:** Classification and distribution of the five type MIR159/319 genes

**Type**	**Definition**			**Number of genes**			**Number of species**		
			
	**D**^**a**^	**seq**^**b**^	**str**^**c**^	**MIR**^**d**^	**can**^**e**^	**all**	**MIR**^**d**^	**can**^**e**^	**all**
			
1	> 60	yes	yes	226	5	231	71	4	71
2	≤ 60	no	no	6	13	19	4	4	6
3	> 60	yes	no	4	0	4	3	0	3
4	> 60	no	yes	5	6	11	4	3	7
5	> 60	no	no	9	3	12	8	2	10

^a ^Distance (number of nucleotides) between miR159/319 and miR159/319* sequences

^b ^Whether two additional well-conserved sequences are present between miR159/319 and miR159/319*

^c ^Whether an extended stem structure without any branch is present.

^d ^Number of collected or identified stem-loops of MIR159/319 genes.

^e ^Number of candidate MIR159/319 genes

The remaining types are with degenerated ACRs (type 3 and type 4) or even lacking these two regions (type 2 and type 5). Type 2 stem-loops appear as in a shortened form without the elongated part probably due to deletion (Table 1 and Figure 2). Sequences of the loop-proximal regions are conserved in type 3 stem-loops (Table 1 and Figure 2). However the secondary structure is disrupted. The elongated stem is maintained in type 4 stem-loops, but sequence similarity in the region corresponding to ACR of type 1 is too weak to be identifiable (Table 1 and Figure 2). Type 5 stem-loops do not have identifiable ACR sequences and the elongated stem is interrupted by multi-loops (Table 1 and Figure 2). Conservation of the type 1 MIR159/319 genes across land plants indicates the origin of MIR159 and MIR319 from a long stem-loop. The type 2 to 5 MIR159/319 genes seems to be variants evolving from type 1. During evolution, the loop-proximal part on their precursor stem-loops may degenerate in sequence and/or structural conservation to different extent. Since the processing of miR159/319 genes depends on correct processing of the loop-proximal duplex [17], these MIR159/319 genes may lose their activity due to unsuccessful processing. For example, the processing of ath-MIR159c, which is a type 4 gene, is far less efficient than that of its paralogues [5]. Likewise, mutations outside of the mature miR319 may lead to failure in the processing of MIR319 in *B.oleracea *[21]. Therefore, some if not all type 2 to 5 MIR159/319 genes might be "pseudo-miRNA" genes unable to produce mature miRNAs.

### Phylogeny of the MIR159/319 genes

During evolution the manner of base substitutions in structured RNA is different from that in protein-coding sequences. The selections for mutations in structured RNA mainly arise from base-pairings to maintain the RNA structures while in protein-coding sequences selection pressure disadvantages mutations in triplet codes that disrupt protein functions through alteration of amino-acids. Based on the structural alignment and consensus RNA secondary structure of the type 1 MIR159/319 genes, we reconstructed the phylogenetic tree using a combination of GTR and doublet models in a Bayesian approach [22]. A well-supported consensus tree was obtained (Additional file 3). The three *A.thaliana *MIR319 paralogues are far more distantly related to each other than that of the ath-MIR159a and ath-MIR159b in the tree, consistent with the inference from the miRNA-carrying segmental duplications in *Arabidopsis *[23]. The observation that ath-MIR159a and ath-MIR159b have very similar expression patterns and are functionally redundant while the three MIR319 paralogues have distinct expression patterns also supports the inferred relative distances among the paralogues of MIR159 and MIR319 in *A.thaliana *[10,14]. In addition, the relationships of ath-MIR319b with its sense and antisense duplicates were correctly resolved in the tree (Additional file 3). These observations provide additional supports for the reliability of the inferred phylogenetic tree.

**Figure 4:**
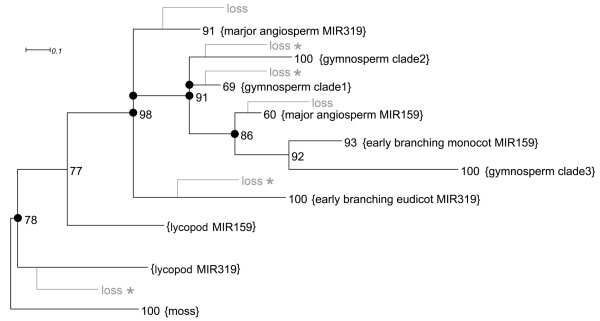
**Condensed phylogenetic tree of type 1 MIR159/319 precursor stem-loops**. The major clades are condensed and indicated in curly brackets. Bayesian posterior probabilities supporting the nodes are indicated by numbers. The deduced gene duplications and losses are indicated by filled circles and gray lines, respectively. The gray lines are schematic and do not indicate the exact point of the losses. Gene losses supported by search of MIR159/319 gene in the entire genome of least one species are indicated by a star. The full tree is shown in Additional file 3.

Using moss, the earliest-branching clade in land plants, as out group, phylogeny of the MIR159/319 could be determined. The MIR159/319 genes from moss, lycopod, gymnosperms and angiosperms can be clearly separated into 10 major clades (Figure 4 and Additional file 3). All extant MIR159/319 genes of moss derive from the earliest branching clade (Figure 4 and Additional file 3). The smo-MIR319 and smo-MIR159 are two extant genes of two clades branching before the emergence of seed plants (Figure 4 and Additional file 3). After the diversification of lycopod MIR319 and MIR159, clades of MIR159/319 specific to either gymnosperm or angiosperm formed (Figure 4 and Additional file 3). In flowering plants, there are two early-branching clades leading to the founding members of MIR159 and MIR319 identified in *A.thaliana *respectively [6,24] (Figure 4 and Additional file 3).

Duplication and loss of genes are deduced from the tree using the criteria that duplications increase the number of paralogues in one clade, while gene losses were inferred by the absence of an ancestral gene in descendant clades. Because the split of orthologous genes must predate or accompany the split of related species that carry them, the time of the duplications relative to the emergence of new plant clades can be deduced. Six duplications can be deduced from the condensed tree showing early-branching clades, one in the common ancestor of vascular plants and five in the common ancestor of seed plants (Figure 4). The tree shows that the split of the MIR319 and MIR159 clades in flowering plants and gymnosperms originated from a series of duplications occurring in the common ancestor of seed plants (Figure 4). This suggests that the MIR319 and MIR159 genes classified by the founding members identified in *A.thaliana *originated from a common ancestor, which may have emerged after the split of moss and lycopod MIR159/319 clades. The two angiosperm MIR319 clades branched earlier than the angiosperm MIR159 clades. Subsequent duplications led to the formation of gymnosperm clades and angiosperm MIR159 clades (Figure 4). Six possible losses in the major clades of MIR159/319 can be deduced from the condensed tree, one in lycopod and five in gymnosperms and angiosperms (Figure 4). Because our analyses do not include all the species in a given clade and entire genomes are only available for a small number of species, the deduced gene losses are incomprehensive and indicate only the possible inclination of gene evolution in the species examined. However, deduced losses are of high confidence in the species for which genome-wide scan of MIR159/319 gene have been performed (see methods). Of the six possible losses, four are supported by the absence of ancestral MIR159/319 genes in one or more species where the entire genomes were examined (Figure 4). The other two losses are deduced to have occurred in gymnosperms whose whole genome sequences are currently not available (Figure 4). Therefore, these two losses and loss of gymnosperm MIR159/319 in 'early branching eudicot MIR319' need to be verified in the future using the whole genomes of gymnosperm species (Figure 4). Considering the current data, these gene losses may have occurred in an interleaved manner that descendant species mutually lost the equivalent of extant genes in other descendant species after gene duplications in their common ancestor. It appears that clade-specific groups of MIR159/319 genes may have formed through the dynamics of gene duplications in ancestors and gene losses in descendants as opposed to the divergence of one ancestral gene into orthologues. The duplications and subsequent losses that occurred before the split of monocots and eudicots are more frequent in the MIR319 clade than the MIR159, indicating that the two clades might have evolved in different ways (Figure 4 and Additional file 3).

### Shift in the proportion of mature MIR159/319 products during ontogeny

**Figure 5:**
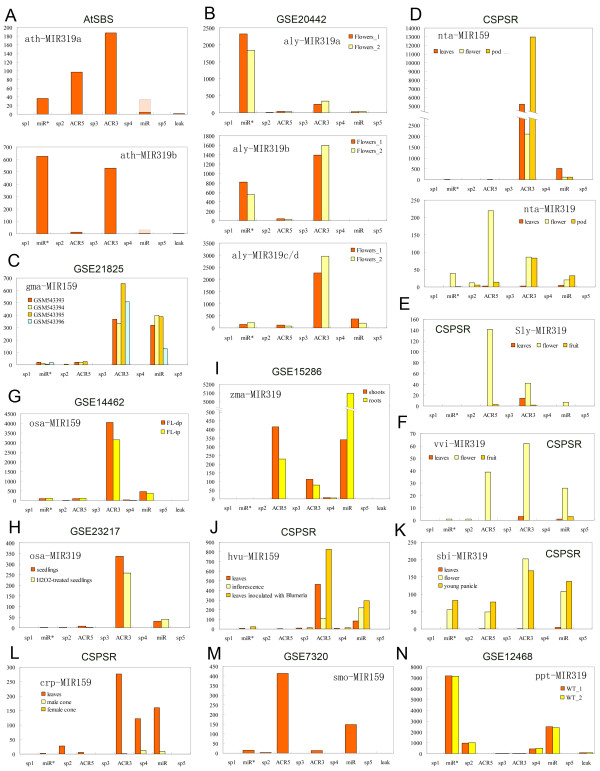
**Non-canonical proportion of MIR159/319 mature products revealed using high throughput sRNA sequencing data**. Normalized sequencing abundances (vertical axis) of mature miRNAs corresponding to each partition (horizontal axis) of the MIR159/319 genes are illustrated. For each database, sequencing abundance was normalized to transcripts per mean of total reads of the samples (listed in Additional file 8). The watermarked parts are reads that also match paralogous genes. Details of the databases are listed in Additional file 8. **A**: Inflorescence tissues of 5-week-old *A.thaliana *plants. **B**: Two replicates of *Arabidopsis lyrata *flowering tissues. **C**: Samples of *Glycine max *shown as accession numbers in NCBI GEO. GSM543393: Richland (I/I, yellow seed) immature seed coats; GSM543394: Williams (i-i/i-i yellow seed) immature seed coats; GSM543395: Williams (i-i/i-i yellow seed) immature cotyledons; GSM543396: Williams mutant 55 (i/i, pigmented seed) immature seed coats. **D**: Tissues from *Nicotiana Tabacum *plants. **E**: Tissues from *Solanum lycopersicum *plants. **F**: Tissues from *Vitis vinifera *plants. **G**: Flag leaves of diploid (FL-dp) and auto-triploid (FL-tp) *O.sativa *plants. **H**: 12-day-old seedlings of *Oryza sativa*. **I**: Shoots and roots of *Z.mays*. Zma-MIR319a/b/c/d are analyzed collectively. **J**: Tissues from *Hordeum vulgare *plants. **K**: Tissues from *Sorghum bicolor *plants. **L**: Tissues from *Cycas rumphii *plants. **M**: *S.moellendorffii *above-ground tissues. **N**: Two biological replicates (WT1, WT2) of 10-day-old protonemata of *P.patens*. The reads for all of the MIR319 genes in moss were included.

MIR159/319 precursors are processed in a phased manner to produce three miRNA duplexes [15,17,18]. We examined the expression of MIR159/319 in 54 publicly available small RNA sequencing databases from 20 species (Additional file 4). Interestingly, the partitions defined by conservation pattern are consistent with the phasing of mature miRNAs from MIR159/319 stem-loops in that very few reads have lower than 80% overlap with any partition (Additional file 4). In most cases, miR159/319 take up the majority of the sequencing reads, while miRNAs from miR*, ACR5 and ACR3 are expressed at much lower levels. The expressions of space partitions are even lower or undetectable (Additional file 4). We refer to this previously identified profile as 'canonical proportions' [17]. However, there are a few non-canonical situations. When we analyze the expressions of MIR159/319 using data from AtSBS [25], we found that in the inflorescence tissues of five-week-old *Arabidopsis thaliana*, the predominant mature products of ath-MIR319a are ACR3 miRNAs, while the ath-MIR319b mainly expresses ACR3 and miR* miRNAs (Figure 5A). In contrast, the ath-MIR159 genes primarily generate miR159 (Additional file 5A). In the flowering tissues of the closely related species *Arabidopsis lyrata*, the non-canonical expression of MIR319 was also supported by two databases [GEO:GSE20442, GEO:GSE20662] from two independent studies using SOLiD and Illumina Genome Analyzer respectively [26,27] (Figure 5B and Additional file 5C). In consensus, expressions of the ACR3 miRNAs are promoted and higher than the miR319 in aly-MIR319c/d, albeit differences in the expressions of aly-MIR319a and aly-MIR319b between the two databases, which is probably due to sampling of the tissues at different stages. In [GEO:GSE20442], the miR319* is significantly higher than miR319 and ACR3 of aly-MIR319a is lower than aly-MIR319b (Figure 5B). In [GEO:GSE20662], miR319a/b is more abundant than miR319a/b* and ACR3 of aly-MIR319a is higher than aly-MIR319b (Additional file 5C). Small RNA sequencing from seed coats and cotyledons of *Glycine max *[GEO:GSE21825] suggests that gma-MIR159 express ACR3 miRNAs at levels similar to or higher than miR159 in these tissues [28] (Figure 5C). In *Nicotiana Tabacum *plants, data from Comparative Sequencing of Plant Small RNAs (CSPSR) showed non-canonical expressions of both MIR159 and MIR319 [29] (Figure 5D). However, in the case of nta-MIR319, the ACR5 miRNA is the most abundant in flower but not in pod (Figure 5D). We also found ACR5-predominant expression of MIR319 in *Solanum lycopersicum *using data from CSPSR [29] (Figure 5E). Again using CSPSR data [29], non-canonical expression of MIR319 was observed in *Vitis vinifera *(Figure 5F).

**Figure 6:**
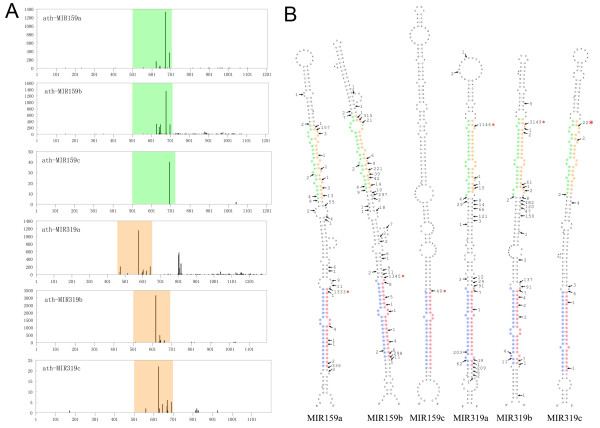
**PARE analysis of the *A. thaliana *MIR159/319 precursor processing**. **A**: Distribution of uncapped 5'RNA end sequencing frequency (vertical axis) in an approximately 1 kb region (horizontal axis) with the precursor stem-loops located at the centre (green for MIR159; orange for MIR319). The major cleavage sites downstream of the ath-MIR319a stem-loop coincide with a reported alternative splicing site [21]. **B**: Detailed PARE-detected cleavage within the MIR159/319 precursor stem-loops. Cleavage sites are indicated by arrows tethered with sequencing abundances supporting the cleavages. The most frequently cleaved site for each MIR is indicated by a red asterisk. The sequences corresponding to the four partitions are colored in the same way as in Figure 2.

Recently, the Parallel Analyses of RNA Ends (PARE) technology was used to detect cleavage products of miRNA targets that contain a phosphate at 5' end by deep sequencing [25,30]. Processing intermediates of miRNA precursor can also be detected by PARE [25]. The differences of miRNA production between MIR159 and MIR319 are also supported by data from *Arabidopsis *PARE database [25] (Figure 6). For MIR319 precursors, the most abundantly sequenced cleavage site was the 5' end of ACR3 miRNAs. However, 5'end of miR159 was the most frequently sequenced site for MIR159 precursors. This coincided with the data from AtSBS (Figure 5A, Figure 6 and Additional file 5A) [25]. Because small RNA sequencing and PARE are different technologies in nature, the observed differential expressions of MIR159 and MIR319 in *Arabidopsis *inflorescence are unlikely to be resulted from biased sequencing. Interestingly, the detected cleavages occur predominantly within the stem-loop pre-miRNAs (Figure 6). This indicates that the MIR159/319 primary transcripts might be processed into miRNA duplexes directly without the preceding step to liberate the stem-loop precursors consistent with the findings of Chekanova et al. [31].

We also observed the switching on of the MIR159 or MIR319 ACR miRNA expression in monocots. In rice, analyses using high throughput small RNA sequencing data deposited under [GEO:GSE14462] suggest that osa-MIR159a mainly produces ACR3 miRNAs in the flag leaves (Figure 5G), which is an organ important for grain filling and protection of the spike from being eroded by pathogens. Sequencing data from [GEO:GSE23217] suggest that ACR3 miRNA is the major product of osa-MIR319 in rice seedlings [32] (Figure 5H). In maize, a shift in the proportion of mature MIR319 products was observed in the tissues of shoots and roots using the data from [GEO:GSE15286] [33] (Figure 5I). Such a transition is not observed for maize MIR159 (Additional file 5H). Another two examples of non-canonical expression of monocot MIR159/319 come from CSPSR data [29] (Figure 5J and 5K). In *Hordeum vulgare *plants, expression level of MIR159 ACR3 miRNA is higher than miR159 in leaves but not in inflorescence tissues, indicating shift of mature product proportions in the two tissues (Figure 5J). In flower and young panicle of *Sorghum bicolor*, ACR3 miRNAs are more abundant than miR319 (Figure 5K). These results indicate that both in eudicots and monocots, the major products of MIR159 or MIR319 genes shift to ACR miRNAs in a highly spatio-temporal specific manner during plant development. This is likely to occur through post-transcriptional mechanisms, because the proportion of the multiple mature products can not be changed solely via transcriptional regulations. In some cases, shift in strand selection also occurred. When sequencing tags can not be unambiguously assigned to a unique MIR159/319 stem-loop, such as maize MIR319 genes in Figure 5I, related paralogues are analyzed collectively. In this case, the observed shift in mature product proportion could possibly be attributed to down- or up-regulation at transcriptional level of different paralogues with fixed mature product proportions. However, there must be a post-transcriptional mechanism to distinguish different paralogues so that they produce multiple mature miRNAs in different proportions.

For the gymnosperms and earlier-branching clades, lycopod and moss, we also analyzed the expressions of the MIR159/319 genes using small RNA sequencing data from CSPSR and [GEO:GSE5103, GEO:GSE7320, GEO:GSE12468] [15,29,34]. In *Cycas rumphii *leaves, the three phased miRNAs at 3' arm, ACR3, sp4 and miR159 are expressed at significant levels, among which ACR3 is the most abundant (Figure 5L). In the lycopod *S.moellendorffii*, the predominant products of both smo-MIR159 and smo-MIR319 are ACR5 miRNAs, consistent with the distance in the inferred phylogenetic tree (Figure 4, Figure 5M and Additional file 5J). The proportion of mature products from the moss MIR319 differs from all other clades in that the ACR miRNAs are rarely expressed while the sp2 and sp4 miRNAs are expressed at significantly higher level (Figure 5N and Additional file 5K). Because the sp2 miRNA of ppt-MIR319d is abundantly expressed, this miRNA is annotated as ppt-miR319d*.2 [15,35,36]. This is consistent with our analysis. In 10-day-old moss protonemata the miR319* are the most abundant products (Figure 5N). The distinct proportion of mature products from the MIR159/319 genes in different clades suggests that the post-transcriptional control of MIR159/319 gene output may have diverged during evolution accounting for the variability of mature miRNA expression profiles of this family.

### Selective up-regulation of mature MIR159/319 miRNAs in siRNA-deficient mutants

**Figure 7:**
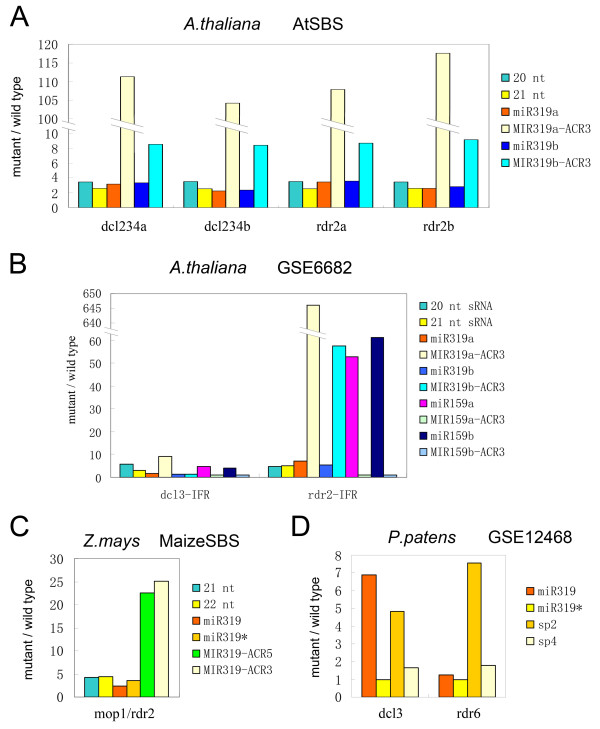
**Selective up-regulation of mature MIR159/319 products in siRNA-deficient mutants revealed using high throughput sRNA sequencing data**. Mutant to wild-type Ratio (Vertical axis) of normalized sequencing abundances of mature MIR159/319 miRNAs or small RNA populations with specific lengths for the indicated mutant plants (Horizontal axis). Species and data accessions are indicated above each plot. Plots of the sequencing abundances are shown in Additional File 6. **A**: Two replicates of *dcl2 dcl3 dcl4 *triple mutants (dcl234a, dcl234b) and two replicates of *rdr2 *mutant plants (rdr2a, rdr2b). All samples are from inflorescence tissues of 5-week-old *Arabidopsis*. **B**: dcl3-IFR, inflorescence tissue of *Arabidopsis dcl3 *mutant plants; rdr2-IFR, inflorescence tissue of *Arabidopsis rdr2 *mutant plants. The ratios for ath-MIR159a and ath-MIR159b ACR3 miRNAs are set to 1 indicating absence or nearly absence of these miRNAs in both wild type and indicated mutants. **C**: Maize small ears 68 days after germination. **D**: 10-day-old *P. patens *protonemata.

In plants, genomic repeats are silenced by approximately 24-nt rasiRNAs whose expression depends on the RDR2 and DCL3 proteins [37,38]. Unexpectedly, we found that in siRNA-deficient mutants of species from eudicot, monocot and moss MIR159/319 miRNAs are selectively up-regulated. Using AtSBS data [25], we found that in the inflorescence tissues of 5-week-old *A.thaliana rdr2 *mutants and *dcl2 dcl3 dcl4 *(*dcl234*) triple mutants, the expressions of the ath-MIR319a and ath-MIR319b ACR3 miRNAs are much higher than the case in wild type, over 100 fold for ath-MIR319a and approximately 8 fold for ath-MIR319b (Figure 7A and Additional file 6A). In contrast, the increased miR319 and miR319* expression is undistinguishable from the enrichment of the 20~21-nt sRNAs in these mutants (Figure 7A). Analyses using data from [GEO:GSE6682] reproduce this phenomenon with the difference that miR159 levels are also up-regulated when the rasiRNA pathway is disrupted (Figure 7B and Additional file 6B) [39]. Consistently in both databases the increased levels for ACR miRNAs of ath-MIR319a are higher than that of ath-MIR319b (Figure 7 A and B). Because the two small RNA sequencing data resources were produced using different methods, 454 for [GEO:GSE6682] and Solexa for AtSBS [25,39], the observed selective up-regulation is unlikely to be bias of sequencing. The selective up-regulation of MIR319 ACR miRNAs is absent in *dcl3 *single mutants (Figure 7B and Additional file 6B), probably due to redundant DCL activities [38,40]. We also examined the expression of MIR159/319 in maize using data from MaizeSBS [41]. Similarly in the small ears (flowers) of maize *mop1*/*rdr2 *mutants, the expression of MIR319 ACR miRNAs is selectively up-regulated (Figure 7C and Additional file 6C). In the *dcl3 *mutant of the moss *P. patens*, the overall abundance of miRNAs does not increase [34]. Using the same small RNA sequencing data, it can be observed that the miR319 increased approximately seven-fold and became the most abundant mature product of MIR319 gene (Figure 7D and Additional file 6D). The sp2 miRNAs also increased more than four-fold (Figure 7D). In *rdr6 *mutants, in which tasiRNAs are depleted without affecting the miRNA abundance [34], only the sp2 miRNAs increased approximately seven-fold (Figure 7D). These results suggest that the accumulation of specific MIR159/319 miRNAs, e.g., the ACR miRNAs in flowering plants, is regulated either directly or indirectly by siRNA pathways at the post-transcriptional level. This connection between the siRNA and miRNA pathways might have an ancient origin.

## Discussion

From the evolutionary perspective, miRNAs are generally classified into highly conserved ancient miRNAs and weakly conserved species- or clade-specific miRNAs [2]. The phylogeny of highly conserved miRNA genes is largely unknown including the ancient miRNA gene family -- MIR319 and MIR159, which play important roles in plant development [5]. The miR319 and miR159 were identified in independent studies using different methods [6,24]. Homologues identified in following studies are classified into two groups according to sequence similarity to the miR319 and miR159 founding members [19]. Although miR159 and miR319 are seemingly related in evolution, because of their similarity in mature miRNA sequence, secondary structure, conservation pattern and biogenesis, their origin is still unclear [5,17]. Evolution of MIR319 genes in closely related *Brassicaceae *species has been studied revealing that the stem-loop and an upstream element of MIR319 are recalcitrant to fast mutations while sequences of other parts are highly variable [21]. We aligned a large number of land plant MIR159/319 stem-loops and reconstructed the phylogenetic tree. Our results support a common origin of MIR159 and MIR319 from two aspects. First, another duplex outside miR159/319 are highly conserved in most MIR159 and MIR319 stem-loops across land plants (Figure 2, Figure 3, Table 1 and Additional file 2). Moreover, partitions defined by the conserved regions are consistent with the phasing of mature MIR159/319 miRNAs from moss to flowering plants [17,18] (Figure 3 and Additional file 4). These observations suggest that MIR159 and MIR319 originated from a common phased stem-loop RNA similar to those discovered in the green alga *Chlamydomonas reinhardtii *and rice recently [42-44]. Second, the inferred phylogenetic tree shows that the MIR159 and MIR319 founding members in *A.thaliana *evolve from two early-branching clades specific to flowering plants, which derived from the common ancestor of seed plant MIR159/319 (Figure 4 and Additional file 3). The tree also shows that the angiosperm MIR319 major clade branched earlier than the angiosperm MIR159 clade (Figure 4). This is consistent with the finding that miR159 is more specialized in target spectrum than the miR319 in *A.thaliana *[5].

The inferred phylogenetic tree reveals the evolutionary history of extant MIR159/319 branches, which might be dated back to the first colonization of land plants about 450 Ma (million years ago). It can be inferred that extensive duplications of MIR159/319 genes might have occurred in the common ancestor of gymnosperms and angiosperms about 370 Ma, in the period of late Devonian to early Carboniferous. These duplications initialized the split of MIR159 and MIR319 in flowering plants. It has been shown that major body-plan innovations during animal phylogeny are concomitant with miRNA repertoire expansions [45]. Hypothesis has been proposed that expansion of miRNAs may contribute to the improvement of body-plan complexity [45,46]. Whether similar scenario is also the case in plants is unknown. From the phylogenetic tree, it can be inferred that an expansion of MIR159/319 family genes might be commitment with the evolution of seed-bearing plants (Figure 4). Recent studies have shown that both miR159 and miR319 play important roles in reproductive growth of flowering plants [8,14], indicating their functional relatedness to the evolution of seed bearing in plants. It has been shown in both plants and animals that miRNA repertoire encoded in the genome is in the dynamics of genesis and loss of miRNA genes during evolution [39,47,48]. Some new born miRNA genes seem to be targetless and evolutionarily transient, likely to serve as resources for the selection of functional miRNAs [39,47]. Similarly, from the phylogenetic tree of MIR159/319 family, we found this ancient miRNA family underwent dynamics of duplication and loss, through which clade or species-specific miRNA gene subgroups have formed. Specifically, during evolution clade-specific subgroups formed in the manner that specific members of the expanded gene family maintained in a given descendant clade were lost in sister clades. Moreover, duplicated gene members maintained in one clade of plants, e.g. miR159 and miR319 in flowering plants, tend to diverge into subfunctionalized groups improving the complexity of miRNA regulation. Similarly, the three members of *Arabidopsis *miR319, which arose from more recent duplications, also diverged into genes with distinct expression patterns and possibly different functions [14].

The ACR miRNAs are much more variable than the miR159 or miR319 (Figure 3), indicating different selection pressure imposed on the two miRNAs on the same stem-loop. Moreover, a small number of MIR159/319 genes (type 2 to 5) lost conservation of the loop-proximal part corresponding to the ACRs of type 1. Since the correct processing of the loop-proximal miRNA duplex is the prerequisite for the dicing of miR159 or miR319 [17], the ACR-lacking MIR159/319 might become pseudo genes unable to produce mature miRNAs like the ath-MIR159c, which can not be efficiently processed when transformed into plants [5]. Alternatively, the expression of non-type-1 MIR159/319 genes might be highly spatio-temporal specific or inducible under specific conditions. Since the non-type 1 MIR159/319 genes are rare and we did not find exact evidence that any species only keep non-type 1 MIR159/319 in their genomes, the non-type 1 MIR159/319 genes might derive from type 1.

With regulatory roles in gene expression, expression of miRNAs is also regulated delicately. Many miRNAs are expressed in specific population of cells dedicated to certain functions during development [1,2]. In animals, growing evidences suggest that miRNA expression is not only regulated at transcriptional level but also at post-transcriptional level. The processing of specific pri-miRNAs by the microprocessor is regulated via diverse mechanisms in various biological processes [49]. Recently, selective stabilization of miRNAs by 3'-terminal adenylation has been reported revealing post-dicing regulation of miRNA abundance by synthesis and degradation homeostasis [50]. However, plant genomes do not possess genes encoding protein factors equivalent to Drosha in animals, which is the RNase III enzyme catalyzing pri- to pre-miRNA cleavage [2]. Question arises whether post-transcriptional mechanisms have also evolved in plants to control the expression of specific miRNAs. Variegated dependencies on miRNA pathway components of miRNA accumulations indicate that there might be miRNA-specific regulations at post-transcriptional steps in plant miRNA biogenesis and stability. For example, it has been reported that null or hypomorphic alleles of DCL1, AGO1, SERRATE, DRB1, ABH1 and CBP20 have different impact on the accumulation of distinct miRNA subsets [51-54]. A family of exoribonucleases responsible for the degradation of plant miRNAs is identified, indicating that mature miRNAs can also be regulated through degradation in plants [55]. We found spatio-temporal specific shifts in the proportion of miRNA products within MIR159/319 stem-loop precursors during development (Figure 5, Figure 6 and Additional file 5). Expression analysis also showed selective up-regulation of mature MIR159/319 miRNAs in siRNA-deficient mutants of moss and flowering plants (Figure 7 and Additional file 6). These observations provide evidences for the evolution of miRNA-specific post-transcriptional regulatory mechanism in plants.

Across the major clades of MIR159/319 genes (Figure 4), the post-transcriptional regulation of miRNA production seems to be diverged. First, general proportions of MIR159/319 mature miRNAs are distinct for the early-branching clades. Moss MIR159/319 genes have pronounced expression in sp2 and sp4 miRNAs. Lycopod MIR159/319 genes produce ACR miRNAs in higher levels than miR159/319 in normal state. The gymnosperm *Cycas rumphii *MIR159 produce three phased miRNAs at comparable levels from 3' arm of the stem-loop precursor in leaves (Figure 5L). In most cases, angiosperm MIR319 and MIR159 generate miR319 or miR159 as major products (Figure 4, Figure 5 and Additional file 4). Second, genes from angiosperm major MIR319 clade and major MIR159 clade can have different proportions of mature miRNAs in specific organs of the same plant (Figure 4, Figure 5 and Additional file 5). Third, genes from different early-branching MIR159/319 clades have different responsiveness to the siRNA deficiency in the mature miRNA expressions (Figure 4 and Figure 7). In addition, paralogues in one early-branching MIR159/319 clade also show differences in tissue-specific proportion of mature miRNAs and responsiveness to siRNA deficiency. For example, the proportion of ath-MIR319a ACR3 miRNA is much higher in 5-week-old inflorescence than that of ath-MIR319b (Figure 5A). The paralogues of MIR319 also showed distinct expressions in *A.lyrata *(Figure 5B and Additional file 5C). In siRNA deficiency mutants, ACR3 miRNA of ath-MIR319a is up-regulated to much higher level than that of ath-MIR319b (Figure 7A and B). Such differences in their expressions are consistent with the inferred phylogenetic distance and different tissue specific expression patterns of ath-MIR319a and ath-MIR319b reported recently [14] (Additional file 3). These observations suggest that the post-transcriptional regulation of MIR159/319 may have specialized during evolution and can serve as another feature that could distinguish their subgroups.

In addition to the small RNAs dedicated to endogenous gene regulation in normal developmental and physiological processes, e.g. miRNA, tasiRNAs, various siRNA pathways have evolved in plants and animals to harness transposons, antagonize infection of pathogens and respond to biotic and abiotic stresses [56,57]. Epigenetic inactivation of transposons and exogenous DNAs mediated by approximately 24-nt siRNAs require the action of DCL3 and RDR2 for their biogenesis and AGO4 in effecter complex [56,57]. DCL2, DCL4 and RDR6 are involved in the production of 21~22-nt siRNAs from virus and *A.tumefaciens *T-DNA, which mediate post-transcriptional silencing of pathogen-derived genes [56,57]. Selective up-regulation of MIR159/319 ACR miRNAs in *rdr2 *and *dcl2 dcl3 dcl4 *triple mutants indicates that intrinsic link between miRNA and the defensive siRNA pathways has evolved in plants (Figure 7). Mutation of the gene encoding RDR2 can cause global changes in small RNA transcriptome and gene expressions including reduction of 24-nt heterochromatic siRNA, enrichment of miRNA and tasiRNAs, reactivation of specific type of transposons and altered expression of genes in chromatin modification pathways [41,58,59]. Some of the miRNAs are up-regulated much higher above the overall enrichment of miRNAs in *rdr2 *mutants, such as the miR163 in *A.thaliana *[41,58]. The up-regulations of MIR159/319 miRNAs in *rdr2 *or *dcl234 *triple mutants are very similar to these cases. However, the up-regulations of MIR159/319 miRNAs are of strong selectivity upon the small RNA products from the same stem-loop suggesting that it is unlikely to be a non-specific process (Figure 7). It was speculated that the different accumulation levels of miRNAs in wild type and *rdr2 *mutants are caused by a secondary level of control by a siRNA-mediated pathway, which depends on RDR2/mop1 [41]. Likewise, the ACR miRNA accumulation of MIR319 genes might be downstream of RDR2-dependent siRNA regulation. Since only ACR miRNAs are induced to high levels while increment of miR319 is similar to the overall enrichment of miRNAs in *rdr2 *or *dcl234 *triple mutants, a post-transcriptional mechanism may distinguish the two miRNA duplexes on the same stem-loop and link the siRNA pathway. Because the selective up-regulation of MIR159/319 genes is observed in both monocot and eudicots, the regulatory link between MIR159/319 genes and siRNA pathway may have an ancient origin. It has been found in *Physcomitrella patens *that silencing of miRNA targets via DNA methylation occurs in PpDCLb mutants and ABA treated wild type plants [60]. This suggests that silencing mechanisms of the heterochromatic siRNA pathway can also be exploited by the miRNA pathway. Our findings revealed another layer of interactions between the two small RNA pathways that production of miRNA can be affected by defects in heterochromatic siRNA pathway.

This uncharacterized mechanism controlling mature product proportion of MIR159/319 genes is distinguished from those previously found in animals in that multiple mature products from a single miRNA stem-loop precursor can be selectively expressed in different situations. PARE data suggest that speed for dicing at the steps during phased processing might be regulated, provided that detection of more intermediate products can serve as the signal for higher dicing speed for the corresponding step of slicing. Accumulations of miR159, miR168 and miR165 are insensitive to decreased DCL1 activity unlike other miRNAs such as miR173, indicative of alternative dicer activity for the processing of these miRNAs [51]. Therefore, it is possible that only processing by alternative factors yields abundant ACR miRNAs but less miR159/319 when alternative activity overrides the canonical. Similar scenarios are possible for other factors involved in miRNA biogenesis and activity, dysfunction of which affect miR159 in a mild way. For example, the ABH1 is required for the processing of some pri-miRNAs but not MIR159 [52,53]. Alternatively, since the theoretical ratio of ACR miRNA to miR159 or miR319 should be one, the variable proportion of MIR159/319 mature products could be resulted from regulation of mature miRNA stability. Probably, which of the MIR159/319 mature miRNAs are incorporated into the RISC and stabilized is tunable. Since the ACR3 miRNA has been shown to associate with AGO4 [17,61], incorporation of ACR3 miRNA into AGO4 might be tremendously increased in the *rdr2 *or *dcl234 *mutants, of which the AGO4 associated siRNAs are depleted [41,58,59]. Increased AGO1 up-regulates miR159 accumulation indicating excessive miR159 might be subjected to active degradation [51]. Likewise, it is possible that the ACR miRNAs might be directly degraded in normal state but stabilized under specific circumstances. This kind of regulation has been reported for a class of 21-nt endogenous siRNAs derived from mRNAs, which accumulated only in the absence of EIN5, a 5'-3' exoribonuclease [53]. Possibly, degradation of processing intermediates could be another way through which maturation of miRNA is regulated. Some miRNA processing intermediates are up-regulated in exosome-deficient mutants, including the loop fragment of MIR159 [31]. Since MIR159/319 precursors are processed in a phased manner, differential exosome-mediated degradation of the intermediates might result in variable product proportions. A combined regulation at multiple steps of miRNA metabolic cycle can not be ruled out. In addition to the unexpected up-regulation of ACR miRNAs, we also observed some cases of higher expression of miR319* than miR319, namely a shift in strand selection (Figure 5A and 5B). The change in strand bias of miR159 and miR159* have been observed in *drb1 *mutant plants, which direct strand selection of miRNA duplexes [54]. Possibly, the spatial-temporal specific selection of strands in miRNA duplexes might be resulted from incongruent expressions of the miRNA and DRB1.

The switching on of the ACR miRNAs in specific tissues and their responsiveness to siRNA deficiency suggests that these miRNAs might be functional. There is only one experimentally verified example that osa-MIR159a ACR3 miRNA induces site-specific cleavage of an mRNA encoding a GT-2-like transcription factor in rice [62]. However, it can not be excluded that the MIR159/319 ACR miRNAs may exert their function over their targets through other mechanisms such as translational repression or RNA mediated DNA methylation [2,60]. In light of the intra-stem-loop regulation, the phased loop-to-base processing of miR159/319 stem-loop precursors may have evolved as framework for the tunable mature miRNA production.

## Conclusions

In this study we showed that MIR159/319 genes with two conserved duplexes in their stem-loops distribute throughout land plants and constitute the most plentiful type classified by conservation pattern. Together with phylogenetic reconstruction and mature miRNA expression analysis, evidences support that the MIR159 and MIR319 have a common origin. Evolution history of this gene family was revealed that the two clades containing the MIR159 and MIR319 founding members identified in *A.thaliana *are specific to flowering plants and originated from duplications occurring before the split of gymnosperm and angiosperms. Interestingly, post-transcriptional regulation of multiple mature miRNAs from some genes of MIR159/319 family has evolved, through which proportion of mature MIR159/319 miRNAs can be changed in a spatio-temporal specific manner or in response to siRNA pathway deficiency. These findings provide explanation for the conserved phased loop-to-base processing of MIR159/319 genes as framework for post-transcriptionally regulated expressions of their mature miRNAs. Taken together, our study provides insights on the evolution of MIR159/319 genes and an unexplored feature of them that the expressions of multiple forms of mature products are post-transcriptionally regulated.

## Methods

### Homology search of miRNA genes

Known MIR159/319 sequences were collected from the miRbase Version 11.0 [19], homologues in EST were identified by Jones-Rhoades and Bartel [7], and the *Brassicaceae *MIR319 sequences were identified by Warthmann *et al*. [21]. In one line, SSEARCH searches were performed against the TIGR Plant Transcript Assembly (http://plantta.tigr.org) and the 11 plant genomes listed in Additional File 7 using ath-miR159a or ath-miR319a as queries [63]. In the other line, all of the mature miRNA sequences of the MIR159/319 family available in miRbase Version 11.0 were used to BLAST search against the nr, gss, htgs and est databases in the NCBI GenBank [64]. The flanking sequences (350-nt or as long as possible) on either side were retrieved for each resulting High-scoring Sequence Pairs (HSP) from the searches. The RNA secondary structures were predicted using RNAfold [65]. The miRNA-like stem-loop structures were selected using modified miRcheck rules [7]. Briefly, 1) the paired bases for miR or miR* (miR: miR159/319 homologous sequence; miR*: antisense sequence in the stem-loop structure corresponding to miR) must be located within one arm, either the 5' or the 3'; 2) there must be no more than 6 unpaired bases in the miR; 3) no more than 3 bulges in the miR; 4) no more than 3-nt differences between the lengths of miR and miR*; 5) no more than 3 asymmetrically unpaired bases in the miR; 6) no more than 3 contiguous unpaired bases in the miR; and 7) the distance between miR and miR* must be no less than 5 and no more than 300 nucleotides. The filtrates were inspected manually to exclude the loose and unstable structures. Finally, if the miR sequence has no more than 3 mismatches or indels compared to a MIR159/319 mature miRNA in the miRbase, the stem-loop was considered a homologue. If the miR sequence contains 4 mismatches or indels, the stem-loop was considered a candidate homologue. Homologous sequences contain more mismatches were not considered. The naming of the new homologues or candidates, 159 or 319, followed the name of the MIR159/319 gene in the miRbase that had the smallest edit distance in the mature miRNA sequence. The abbreviations for the species names are: afo: *Aquilegia formosa*; ace: *Arabidopsis cebennensis*; aha: *Arabidopsis halleri*; aly: *Arabidopsis lyrata subsp. Petraea*; ath: *Arabidopsis thaliana*; ahy: *Arachis hypogaea*; bvl: *Barbarea vulgaris*; bvu: *Beta vulgaris*; bst: *Boechera stricta*; bna: *Brassica napus*; bol: *Brassica oleracea*; bra: *Brassica rapa subsp*. *Pekinensis*; cbp: *Capsella bursa-pastoris*; cru: *Capsella rubella*; cso: *Centaurea solstitialis*; cch: *Cheiranthus cheiri*/*Erysimum cheiri*; ccl: *Citrus clementina*; csi: *Citrus sinensis*; csu: *Citrus sunki*; cca: *Coffea canephora*; cor: *Conringia orientalis*; cme: *Cucumis melo subsp. Melo*; crp: *Cycas rumphii*; dso: *Descurainia sophia*; dka: *Diospyros kaki*; ees: *Euphorbia esula*; far: *Festuca arundinacea*; fve: *Fragaria vesca*; gma: *Glycine max*; htu: *Helianthus tuberosus*; hvu: *Hordeum vulgare*; ini: *Ipomoea nil*; lsl: *Lactuca saligna*; lst: *Lactuca sativa*; lse: *Lactuca serriola*; ltu: *Liriodendron tulipifera*; lja: *Lotus japonicus*; mma: *Malcolmia maritima*; mdo: *Malus × domestica*; mes: *Manihot esculenta*; mtr: *Medicago truncatula*; Nof: *Nasturtium officinale*; nta: *Nicotiana tabacum*; oal: *Oryza alta*; oco: *Oryza coarctata*; ogl: *Oryza glaberrima*; omi: *Oryza minuta*; oni: *Oryza nivara*; opu: *Oryza punctata*; ori: *Oryza ridleyi*; oru: *Oryza rufipogon*; osa: *Oryza sativa*; osg: *Oryza sativa × Oryza glaberrima*; pvi: *Panicum virgatum*; pgl: *Pennisetum glaucum*; pvu: *Phaseolus vulgaris*; ppt: *Physcomitrella patens*; pga: *Picea glauca*; psi: *Picea sitchensis*; pta: *Pinus taeda*; ptf: *Poncirus trifoliata*; pde: *Populus deltoides*; pni: *Populus nigra*; ptr: *Populus tremula*; ptt: *Populus tremula × Populus tremuloides*; ptc: *Populus trichocarpa*; rra: *Raphanus raphanistrum subsp. Raphanistrum*; rco: *Ricinus communis*; rin: *Rorippa indica*; sof: *Saccharum officinarum*; smo: *Selaginella moellendorffii*; svi: *Sibara virginica*; sly: *Solanum lycopersicum*; sbi: *Sorghum bicolor*; tar: *Thlaspi arvense*; tae: *Triticum aestivum*; vun: *Vigna unguiculata*; vvi: *Vitis vinifera*; zma: *Zea mays*. This three-letter code shown in all lower case is used for sequences from miRbase [66], all upper case letters are used for sequences from Jones-Rhoades and Bartel [7], and others are written with only the first letter capitalized.

### Taxonomic tree

The taxonomic tree was plotted using NCBI taxonomy (http://www.ncbi.nlm.nih.gov/Taxonomy/CommonTree/wwwcmt.cgi) and drawn by NJplot [67].

### Structural alignment and phylogenetic inference

All of the stem-loop sequences of the MIR159 family were aligned using the software T-coffee version 6.06 [68]. Sequences that can't be aligned correctly in the conserved regions outside of miR159/319 and miR159/319* were excluded in subsequent analysis. The remaining sequences were aligned again. Secondary structures were produced for each sequence in the alignment using RNAfold [65]. The software 4SALE was then used to correct the alignment manually by considering the agreement in secondary structure [69]. After removing the columns that contain more than a 75% gap, the consensus sequences and structures were produced using ConStruct, RNAalifold, alidot and SARSE [70-73]. Structure logos were produced using slogo [74]. The partitions of the well-aligned stem-loops were divided empirically by assigning approximately 21-nt for the well-conserved regions. The phylogenetic tree was inferred using MrBayes version 3.1 with the GTR model for unpaired regions and the Doublet model for paired regions with 4 discrete gamma distribution categories [22]. Two chains were run for 20 million generations. Trees were sampled every 100 generations. The consensus tree was summarized using a 50 majority rule from 300,000 trees after removing the burn-in. Only one sequence was retained for identical sequences in the alignment for phylogenetic inference. These representative taxa were replaced with nodes of the original redundant taxa, for which posterior probabilities were set to 100 and branch lengths were set to 0.001 for viewing. The phylogenetic tree was edited using Dendroscope and MEGA4 [75,76]. Gene duplications were deduced from the tree using the criteria that duplications increase the number of paralogues in one clade, while gene losses were inferred by the absence of an ancestral gene in descendant clades.

### Re-analysis of existing small RNA sequencing data

Small RNA sequences were mapped on the stem-loop sequences using a customized perl script. Only perfectly matched small RNAs were considered. The small RNA sequencing resources are listed in Additional File 8. Sequencing abundances were normalized to the average sequencing scale for all samples in a given database unless the values had already been normalized using the original data generators. The expression value for a specific partition in the alignment was calculated by summing all the normalized sequencing frequencies of unique small RNAs that had 80% or more of their nucleotides overlap with that partition. The small RNAs that could not be assigned to any partition are categorized as "leak" to denote that these mature miRNAs were inconsistent with the phased processing. The mutant-to-wild type ratios were calculated using the maximum possible values, which included reads that also matched the closely related paralogues when an individual miRNA gene was studied. For [GEO:GSE12468] of *P. patens *[34], the mutant-to-wild type ratio was calculated by using the mean of two replicates for each of the wild type and mutant values.

### PARE (Parallel Analysis of RNA Ends) data analysis

One part of PARE sequence tags was downloaded from the AtPARE database (http://mpss.udel.edu/at_pare). The other part was obtained from [GEO:GSE11007] [30]. Sequencing abundances for all samples were normalized to TP10M (transcript per 10 million reads). The tags that represent uncapped 5' RNA ends were mapped to the *Arabidopsis thaliana *MIR159 and MIR319 genes, stem-loops or an approximately 1 kb sequence with the stem-loop at the centre. The pri-MIR319a is from [GenBank:AY922324] [21]. The frequency of the cleavage between two nucleotides was calculated by summing the sequencing abundances of all the sequence tags whose 5' first nucleotide situated exactly at the second nucleotide. Total cleavages were calculated by summing the values of all the samples for a cleavage site. The maximum possible frequencies that included tags that also matched closely related homologues were used in Figure 6. RNA secondary structures were drawn using XRNA (http://rna.ucsc.edu/rnacenter/xrna/xrna.html).

## List of Abbreviations

miRNA: microRNA; ACR: alternative conserved region; RISC: RNA induced silencing complex

## Authors' contributions

YL conceived and designed the research, performed the analysis and wrote the paper. CL participated in the analysis. GD helped with the phylogenetic reconstruction. YJ supervised the project and wrote the paper. All authors read and approved the final manuscript.

## Supplementary Material

Additional file 1 **Sequences of the MIR159/319 stem-loops**. All the sequences are in DNA form in FASTA format in a plain text file. Click here for file

Additional file 2 **Structural alignment of the 231 type 1 MIR159/319 precursor stem-loops**. The structural annotations are output from ConStruct. Background colours: green for loops, red for consensus base pairs, pink for co-varying pairs; white for non-base pairs in paired regions. The abbreviations for the species are in Methods section. Click here for file

Additional file 3 **Reconstructed phylogenetic tree of type 1 MIR159/319 genes**. The Bayesian posterior probabilities are indicated by numbers. The major clades of land plants are highlighted: red, eudicots; green, monocots; light blue, other flowering plants; blue, gymnosperms; orange, lycopod; gray, moss. The deduced duplications before the split of monocots and eudicots and related losses are indicated by filled circles and gray lines, respectively. Two ath-MIR319b redundant sequences, Ath-MIR319d (sense) and Ath-MIR319e (antisense), which are enclosed in squares, were joined in the phylogenetic inference. They are clustered together in the tree, and their relationships have been correctly resolved. Repeated runs generated the identical topology with slight differences in the posterior probability and branch lengths. The abbreviations for the species are provided in the Methods. Click here for file

Additional file 4 **Overall expression profile of the nine partitions for type 1 MIR159/319 genes**. The normalized values of sequencing abundances for each partition are listed in a table. Click here for file

Additional file 5 **More examples of the proportion of mature products from MIR159/319 genes**. Vertical axis: normalized sequencing abundances of small RNAs; horizontal axis: partitions of the MIR159/319 stem-loops. Normalizations are the same as in Figure 5. **A-B and D-J**: Related databases and series are the same as in Figure 5. **C**: Mature miRNA expressions of MIR159 and MIR319 from *Arabidopsis lyrata *leaves and two replicates of flowers stage 1-12. **E**: Proportion of mature miRNA from moss MIR319 genes. The color-coded series are: Pt, 7-day-old protonemata; PG, 14-day-old protonemata and young gametophores; GS, 60-day-old gametophores and sporophytes.. Click here for file

Additional file 6 **Comparison of sequencing abundances of MIR159/319 miRNAs in wild type and siRNA-deficient mutants**. The vertical axis indicates the sequencing abundances, and the horizontal axis indicates partitions of MIR159/319 stem-loops. Watermarked parts are reads that also match paralogous genes. The color-coded series indicate the sources of tissue and the genotypes used to construct the small RNA libraries. **A**: WT, wild type; dcl234a and dcl234b, two replicates of *dcl2 dcl3 dcl4 *triple mutants; rdr2a and rdr2b, two replicates of *rdr2 *mutants. **B**: IFR, inflorescence; LF, leaves; SD, seedling; P1,P3, leaves inoculated with pseudomonas for 1 or 3 hours; mixed, tags with unidentifiable barcodes. **C**: WT, wild type; mop1, *mop1*/*rdr2 *mutants. **D**: WT_1 and WT_2, two replicates of wild type moss; *dcl3-5 *and *dcl3-10*, two lines of dcl3 mutants; rdr6_1 and rdr6_2, two replicates of *rdr6 *mutants. Click here for file

Additional file 7 Genome resources used for homology search Click here for file

Additional file 8 Small RNA sequencing databases used for MIR159/319 expression analysis Click here for file
