# Supplementary figures and images for: Evolution of MIR159/319 microRNA genes and their post-transcriptional regulatory link to siRNA pathways
Source: BMC Evol Biol. 2011 May 12;11:122. doi: 10.1186/1471-2148-11-122 (PMC3118147; doi:10.1186/1471-2148-11-122)

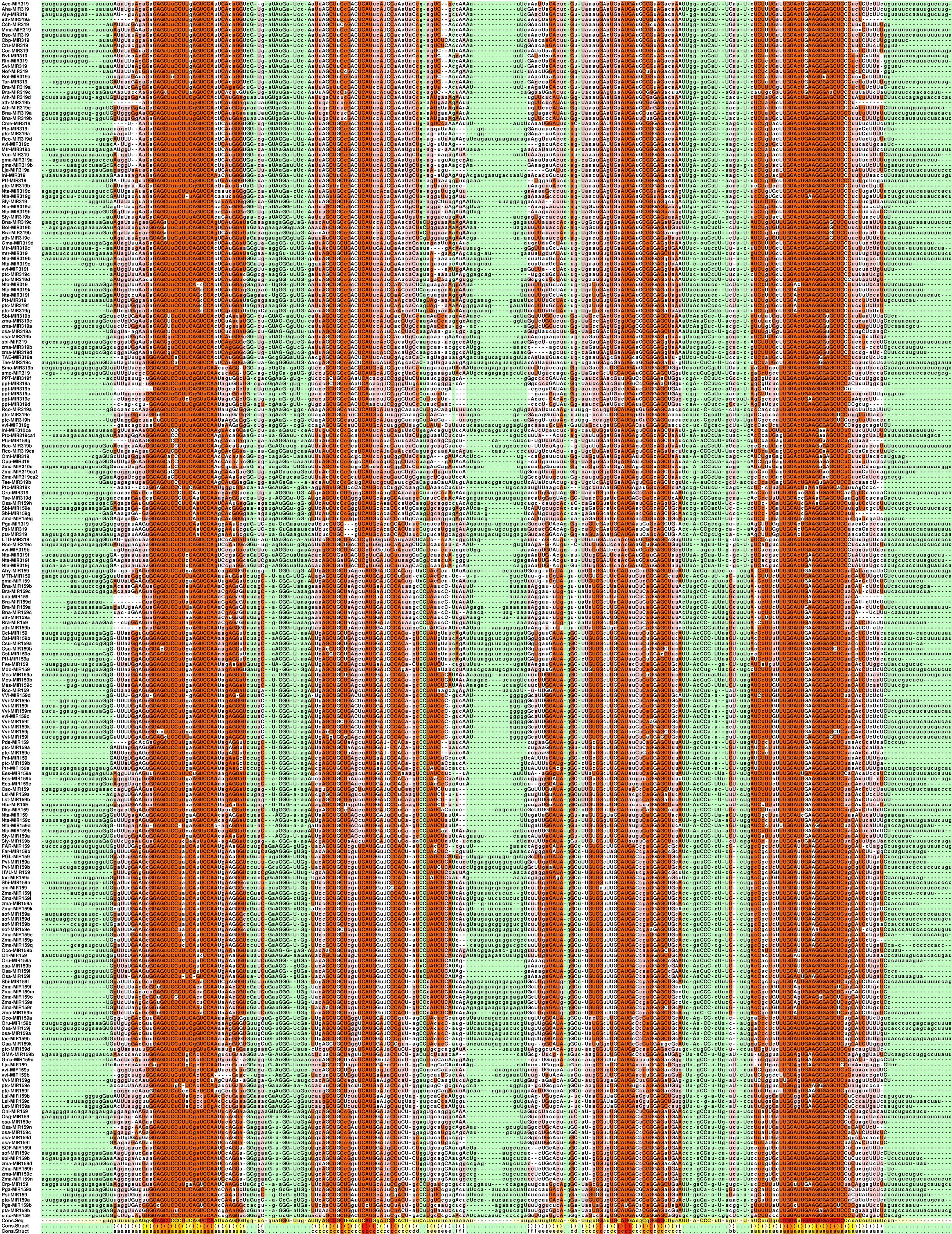

Supplement: Additional file 2 — Structural alignment of the 231 type 1 MIR159/319 precursor stem-loops. The structural annotations are output from ConStruct. Background colours: green for loops, red for consensus base pairs, pink for co-varying pairs; white for non-base pairs in paired regions. The abbreviations for the species are in Methods section. [file 1471-2148-11-122-S2.JPEG]

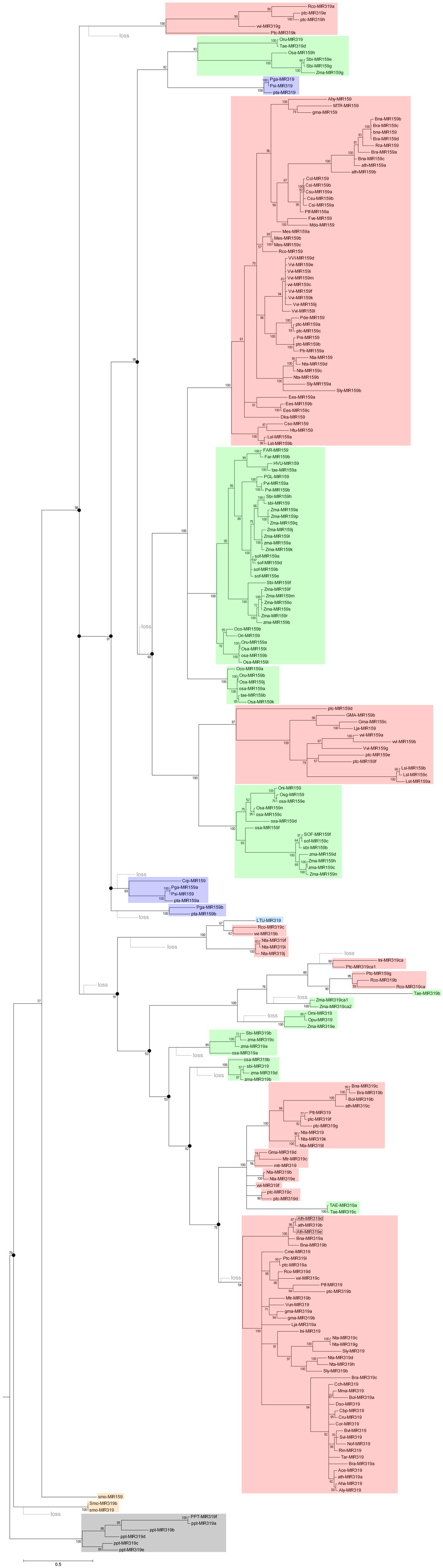

Supplement: Additional file 3 — Reconstructed phylogenetic tree of type 1 MIR159/319 genes. The Bayesian posterior probabilities are indicated by numbers. The major clades of land plants are highlighted: red, eudicots; green, monocots; light blue, other flowering plants; blue, gymnosperms; orange, lycopod; gray, moss. The deduced duplications before the split of monocots and eudicots and related losses are indicated by filled circles and gray lines, respectively. Two ath-MIR319b redundant sequences, Ath-MIR319d (sense) and Ath-MIR319e (antisense), which are enclosed in squares, were joined in the phylogenetic inference. They are clustered together in the tree, and their relationships have been correctly resolved. Repeated runs generated the identical topology with slight differences in the posterior probability and branch lengths. The abbreviations for the species are provided in the Methods. [file 1471-2148-11-122-S3.JPEG]

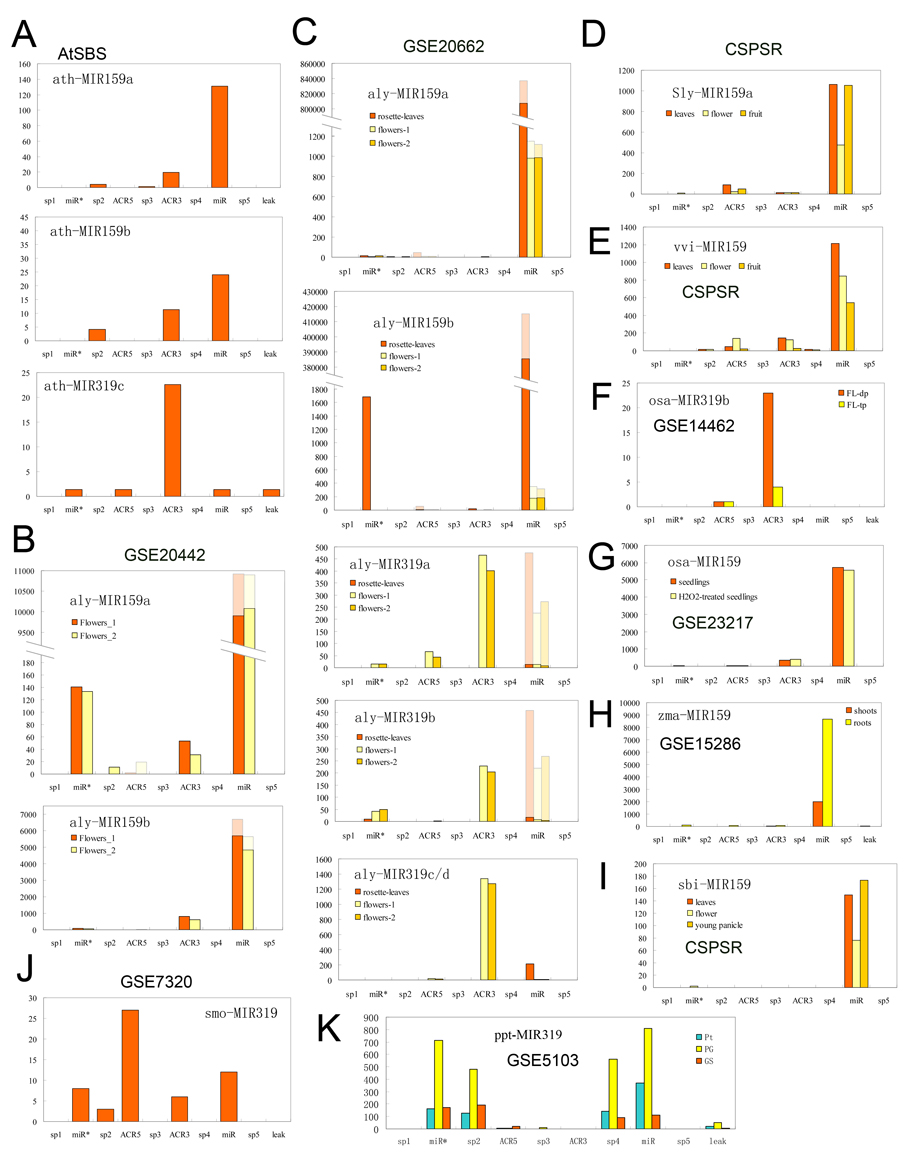

Supplement: Additional file 5 — More examples of the proportion of mature products from MIR159/319 genes. Vertical axis: normalized sequencing abundances of small RNAs; horizontal axis: partitions of the MIR159/319 stem-loops. Normalizations are the same as in Figure 5. A-B and D-J: Related databases and series are the same as in Figure 5. C: Mature miRNA expressions of MIR159 and MIR319 from Arabidopsis lyrata leaves and two replicates of flowers stage 1-12. E: Proportion of mature miRNA from moss MIR319 genes. The color-coded series are: Pt, 7-day-old protonemata; PG, 14-day-old protonemata and young gametophores; GS, 60-day-old gametophores and sporophytes.. [file 1471-2148-11-122-S5.JPEG]

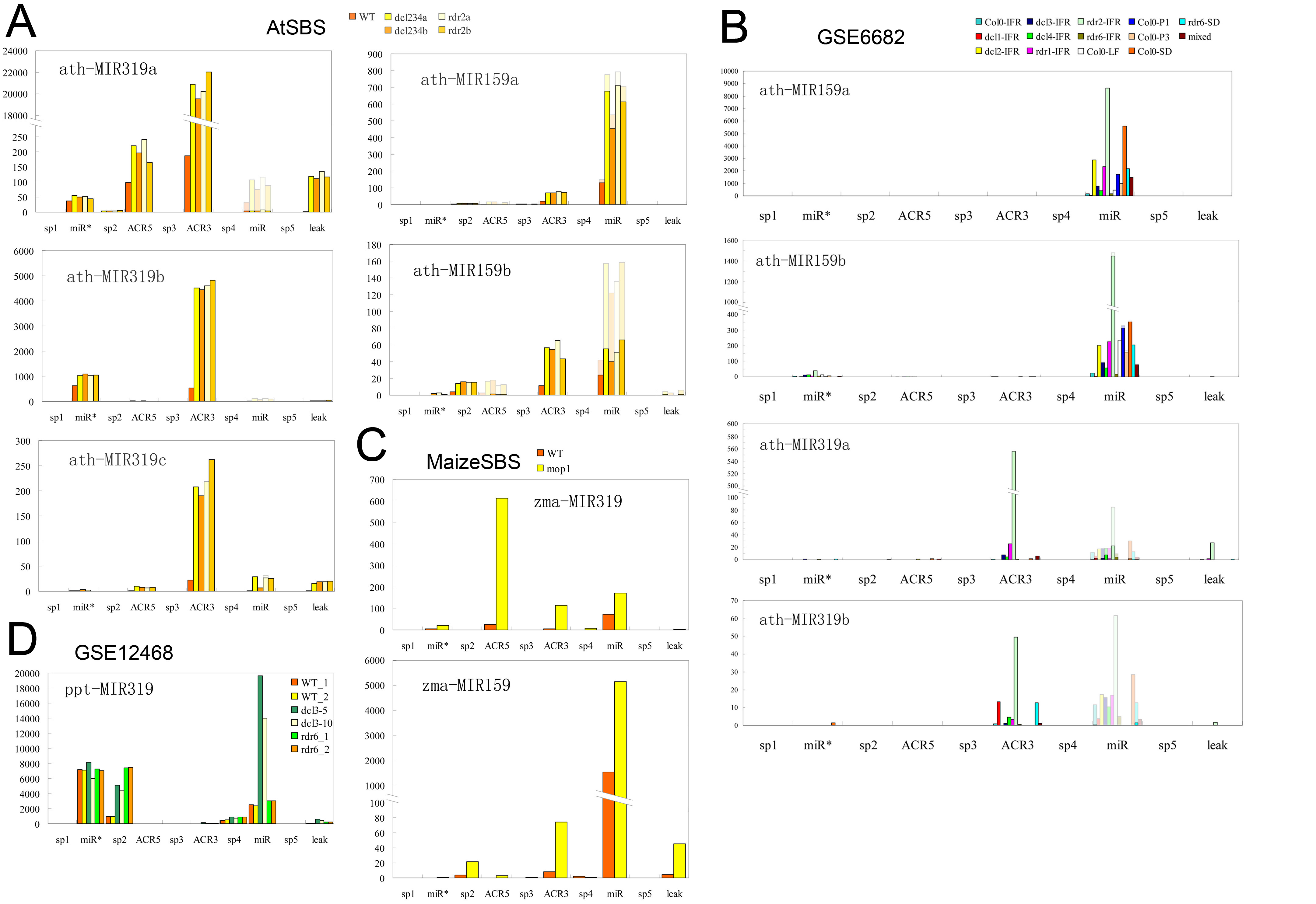

Supplement: Additional file 6 — Comparison of sequencing abundances of MIR159/319 miRNAs in wild type and siRNA-deficient mutants. The vertical axis indicates the sequencing abundances, and the horizontal axis indicates partitions of MIR159/319 stem-loops. Watermarked parts are reads that also match paralogous genes. The color-coded series indicate the sources of tissue and the genotypes used to construct the small RNA libraries. A: WT, wild type; dcl234a and dcl234b, two replicates of dcl2 dcl3 dcl4 triple mutants; rdr2a and rdr2b, two replicates of rdr2 mutants. B: IFR, inflorescence; LF, leaves; SD, seedling; P1,P3, leaves inoculated with pseudomonas for 1 or 3 hours; mixed, tags with unidentifiable barcodes. C: WT, wild type; mop1, mop1/rdr2 mutants. D: WT_1 and WT_2, two replicates of wild type moss; dcl3-5 and dcl3-10, two lines of dcl3 mutants; rdr6_1 and rdr6_2, two replicates of rdr6 mutants. [file 1471-2148-11-122-S6.JPEG]
