# Supplementary material for: Evolution of MIR159/319 microRNA genes and their post-transcriptional regulatory link to siRNA pathways
Source: BMC Evol Biol. 2011 May 12;11:122. doi: 10.1186/1471-2148-11-122 (PMC3118147; doi:10.1186/1471-2148-11-122)
Supplement: Additional file 4 — Overall expression profile of the nine partitions for type 1 MIR159/319 genes. The normalized values of sequencing abundances for each partition are listed in a table. [file 1471-2148-11-122-S4.DOC]

| **Additional file 4**. Overall expression profile of the nine partitions for type 1 MIR159/319 genes | | | | | | | | | | | | |
| --- | --- | --- | --- | --- | --- | --- | --- | --- | --- | --- | --- | --- |
|  |  |  | Normalized Sequencing Abundance ^b^ | | | | | | | | | |
| Species | Database ^a^ |  | Sp1 | miR* | Sp2 | ACR5 | Sp3 | ACR3 | Sp4 | miR | Sp5 | leak |
| ath | GSE5228 |  | 0 | 120 | 11 | 66 | 2 | 10 | 0 | 8283 | 0 | 0.01 % |
| ath | GSE6682 |  | 0 | 107 | 0 | 20 | 0 | 699 | 0 | 31564 | 0 | 0.09 % |
| ath | At454 |  | 0 | 21 | 0 | 8 | 0 | 2 | 0 | 1699 | 0 | 0.06 % |
| ath | AtSBS |  | 0 | 7236 | 132 | 1230 | 18 | 105250 | 3 | 4513 | 0 | 0.61 % |
| ath | AtSC |  | 0 | 82 | 13 | 13 | 1 | 699 | 0 | 240425 | 0 | 0.00 % |
| ath | GSE10036 |  | 0 | 480 | 1 | 42 | 0 | 1241 | 0 | 79506 | 0 | 0.03 % |
| ath | GSE10180 |  | 0 | 48 | 0 | 21 | 2 | 49 | 0 | 4410 | 0 | 0.02 % |
| ath | GSE12037 |  | 0 | 1046 | 10 | 125 | 1 | 32 | 0 | 288564 | 0 | 0.00 % |
| ath | GSE13419 |  | 0 | 0 | 2 | 1 | 0 | 2 | 0 | 1590 | 0 | 0.18 % |
| ath | GSE13605 |  | 0 | 10318 | 52 | 436 | 14 | 264 | 25 | 4248777 | 0 | 0.00 % |
| ath | GSE14696 |  | 0 | 2380 | 31 | 141 | 0 | 241 | 6 | 2210060 | 0 | 0.00 % |
| ath | GSE15348 |  | 0 | 11 | 3 | 1 | 0 | 14 | 0 | 489 | 0 | 0.19 % |
| ath | GSE15443 |  | 0 | 18 | 2 | 47 | 1 | 45 | 0 | 10665 | 0 | 0.00 % |
| ath | GSE6478 |  | 0 | 6 | 0 | 23 | 0 | 39 | 0 | 15585 | 0 | 0.02 % |
| mtr | GSE13761 |  | 0 | 8 | 1 | 6 | 0 | 110 | 0 | 92387 | 0 | 0.02 % |
| mtr | GSE15438 |  | 0 | 0 | 0 | 3 | 0 | 52 | 0 | 5919 | 0 | 0.00 % |
| aly | GSE18077 |  | 0 | 78 | 2 | 21 | 0 | 9 | 0 | 5850 | 0 | 0.00 % |
| aly | GSE20442 |  | 0 | 6293 | 22 | 355 | 0 | 10309 | 3 | 32964 | 0 | 0.02 % |
| aly | GSE20662-454 |  | 0 | 11 | 0 | 25 | 0 | 36 | 0 | 11179 | 0 | 0.00 % |
| aly | GSE20662-G1A |  | 0 | 1851 | 15 | 208 | 0 | 3949 | 0 | 1225911 | 0 | 0.00 % |
| csi | CSPSR |  | 0 | 21 | 1 | 7 | 1 | 50 | 0 | 1938 | 0 | 0.00 % |
| csi | GSE18207 |  | 0 | 3 | 0 | 1 | 0 | 3 | 0 | 698 | 0 | 0.00 % |
| gma | GSE21825 |  | 0 | 56 | 6 | 70 | 0 | 1870 | 0 | 1239 | 0 | 0.00 % |
| lst | CSPSR |  | 0 | 35 | 26 | 0 | 0 | 56 | 84 | 1478 | 0 | 0.00 % |
| nta | CSPSR |  | 0 | 69 | 35 | 263 | 0 | 20457 | 6 | 811 | 0 | 0.27 % |
| osa | CSRDB |  | 0 | 5 | 0 | 2 | 0 | 8 | 1 | 263 | 0 | 0.00 % |
| osa | GSE11014-454 |  | 0 | 138.27 | 0 | 46.09 | 0 | 110.23 | 0 | 1065.74 | 0 | 0.00 % |
| osa | GSE11014-G1A |  | 0.29 | 16.07 | 1.46 | 12.55 | 0 | 126.11 | 4.37 | 37.39 | 0 | 0.15 % |
| osa | MyRNA |  | 0 | 5 | 0 | 1 | 0 | 1 | 0 | 3 | 0 | 0.00 % |
| osa | GSE12317 |  | 0 | 5 | 0 | 0 | 0 | 17 | 2 | 56 | 0 | 0.00 % |
| osa | GSE13152 |  | 0 | 15 | 0 | 56 | 0 | 71 | 13 | 451 | 0 | 0.16 % |
| osa | GSE14462 |  | 0 | 217 | 18 | 231 | 0 | 7235 | 48 | 865 | 0 | 0.02 % |
| osa | GSE16350 |  | 0 | 36 | 15 | 28 | 0 | 54 | 22 | 9110 | 0 | 0.08 % |
| osa | GSE18250 |  | 0 | 919 | 1 | 20 | 0 | 102 | 250 | 3408914 | 0 | 0.00 % |
| osa | GSE23217 |  | 0 | 33 | 2 | 43 | 3 | 1314 | 1 | 11351 | 0 | 0.00 % |
| tae | CSPSR |  | 0 | 6 | 0 | 6 | 0 | 118 | 4 | 558 | 0 | 0.14 % |
| hvu | CSPSR |  | 0 | 32 | 1 | 3 | 23 | 1399 | 20 | 598 | 0 | 0.00 % |
| sbi | CSPSR |  | 0 | 140 | 0 | 128 | 0 | 371 | 0 | 650 | 0 | 0.08 % |
| pvi | CSPSR |  | 0 | 1 | 0 | 14 | 0 | 275 | 0 | 1812 | 0 | 0.57 % |
| pine ^c^ | MyRNA |  | 0 | 2 | 0 | 0 | 0 | 0 | 0 | 2 | 0 | 0.00 % |
| crp | CSPSR |  | 0 | 2 | 28 | 6 | 0 | 278 | 134 | 169 | 0 | 0.00 % |
| ppt | GSE5103 |  | 0 | 1042 | 804 | 30 | 10 | 0 | 794 | 1289 | 0 | 1.97 % |
| ppt | GSE12468 |  | 0 | 42752 | 26404 | 44 | 69 | 434 | 4277 | 44500 | 0 | 1.45 % |
| ptc | Barakat et al. |  | 0 | 2 | 0 | 0 | 0 | 3 | 0 | 1088 | 0 | 0.30 % |
| ptc | CSPSR |  | 0 | 9 | 2 | 35 | 0 | 231 | 1 | 4280 | 0 | 0.26 % |
| sly | GSE12081 |  | 0 | 17 | 0 | 30 | 0 | 5 | 0 | 896 | 0 | 0.00 % |
| sly | CSPSR |  | 0 | 10 | 0 | 302 | 0 | 95 | 0 | 2598 | 0 | 1.18 % |
| vvi | GSE18450 |  | 0 | 61 | 28 | 420 | 17 | 3142 | 0 | 924505 | 0 | 0.00 % |
| vvi | CSPSR |  | 0 | 5 | 32 | 246 | 2 | 360 | 27 | 2636 | 0 | 0.24 % |
| smo | GSE7320 |  | 0 | 23 | 3 | 441 | 0 | 20 | 0 | 160 | 0 | 0.00 % |
| zma | CSRDB |  | 0 | 5 | 0 | 0 | 0 | 5 | 0 | 49 | 0 | 0.00 % |
| zma | MaizeSBS |  | 0 | 26 | 25 | 644 | 1 | 200 | 12 | 6954 | 0 | 0.66 % |
| zma | GSE15286 |  | 0 | 132 | 15 | 722 | 0 | 321 | 14 | 16134 | 0 | 0.19 % |
| zma | CSPSR |  | 0 | 22 | 31 | 275 | 0 | 125 | 4 | 3934 | 0 | 0.27 % |
| 1. Small RNA sequence databases used to map on the stem-loop sequences of MIR159 family genes. 2. Notice that values between different databases are not comparable because the scales of the sequencing vary and benchmarks for normalization are different. The “leak” column is the percent of leak value in the total value of all the small RNAs that were mapped on the MIR159/319 family stem-loops. 3. The “pine” includes *Pinus teada*, *Picea glauca* and *Picea sitchensis*，and sRNAs were from *Pinus concorta*. | | | | | | | | | | | | |
