# Supplementary material for: Evolution of MIR159/319 microRNA genes and their post-transcriptional regulatory link to siRNA pathways
Source: BMC Evol Biol. 2011 May 12;11:122. doi: 10.1186/1471-2148-11-122 (PMC3118147; doi:10.1186/1471-2148-11-122)
Supplement: Additional file 7 — Genome resources used for homology search [file 1471-2148-11-122-S7.DOC]

| **Additional file 7** Genome resources used for homology search | | |
| --- | --- | --- |
| Common name | Latin name | URL^a^ |
| maize | *Zea mays* | <http://maize.tigr.org/release5.0/azm5.shtml> |
| black cottonwood | *Populus trichocarpa* | <http://genome.jgi-psf.org/Poptr1_1/Poptr1_1.home.html> |
| sorghum | *Sorghum bicolor* | <http://www.phytozome.net/sorghum> |
| grapevine | *Vitis vinifera* | <http://www.genoscope.cns.fr/externe/English/Projets/Projet_ML/index.html> |
| barrel medic | *Medicago truncatula* | <http://www.medicago.org/genome/downloads/Mt2/> |
| castor bean | *Ricinus communis* | <http://castorbean.tigr.org/> |
| lycopod | *Selaginella moellendorffii* | <http://genome.jgi-psf.org/Selmo1/Selmo1.home.html> |
| moss | *Physcomitrella patens* | <http://genome.jgi-psf.org/Phypa1_1/Phypa1_1.home.html> |
| tomato | *Solanum lycopersicum* | <ftp://ftp.sgn.cornell.edu/tomato_genome/> |
| green alga | *Chlamydomonas reinhardtii* | <http://genome.jgi-psf.org/Chlre3/Chlre3.home.html> |
| - | *Volvox carteri* | http://genome.jgi-psf.org/Volca1/Volca1.home.html |
| ^a^ Uniform Resource Locator | | |
