# Supplementary material for: Evolution of MIR159/319 microRNA genes and their post-transcriptional regulatory link to siRNA pathways
Source: BMC Evol Biol. 2011 May 12;11:122. doi: 10.1186/1471-2148-11-122 (PMC3118147; doi:10.1186/1471-2148-11-122)
Supplement: Additional file 8 — Small RNA sequencing databases used for MIR159/319 expression analysis [file 1471-2148-11-122-S8.DOC]

| **Additional file 8**. Small RNA sequencing databases used for MIR159/319 expression analysis | | | |
| --- | --- | --- | --- |
| Species | Database^a^ | URL^b^ | Standard for normalization^c^ |
| *A．thaliana* | GSE5228 | NCBI GEO | 221817 |
|  | GSE6682 | NCBI GEO | 33601 |
|  | AtSBS | http://mpss.udel.edu/at_sbs/ | 2358618 |
|  | AtMPSS-454 | http://mpss.udel.edu/at/ | 11674 |
|  | AtSC | http://chromatin.cshl.edu/epiculture/ | 3796566 |
|  | GSE6478 | NCBI GEO | 74859 |
|  | GSE10036 | NCBI GEO | 710192 |
|  | GSE10180 | NCBI GEO | 58597 |
|  | GSE12037 | NCBI GEO | 812468 |
|  | GSE13419 | NCBI GEO | 54316 |
|  | GSE13605 | NCBI GEO | 2867460 |
|  | GSE14696 | NCBI GEO | 1244205 |
|  | GSE15348 | NCBI GEO | 402444 |
|  | GSE15443 | NCBI GEO | 81200 |
| *A.lyrata* | GSE18077 | NCBI GEO | NA |
|  | GSE20442 | NCBI GEO | 45089364 |
|  | GSE20662 | NCBI GEO | 134776 (454) / 4381086 (G1) |
| *O．sativa* | CSRDB | http://sundarlab.ucdavis.edu/smrnas/ | NA |
|  | MyRNA | ftp://ftp03.bcgsc.ca/public/pine_rice_smRNA/ | NA |
|  | GSE11014 | NCBI GEO | 1000000 |
|  | GSE12317 | NCBI GEO | 104741 |
|  | GSE13152 | NCBI GEO | 217620 |
|  | GSE14462 | NCBI GEO | 6654113 |
|  | GSE16350 | NCBI GEO | 96793 |
|  | GSE18250 | NCBI GEO | 3235708 |
|  | GSE23217 | NCBI GEO | 3943775 |
| *Z．mays* | CSRDB | http://sundarlab.ucdavis.edu/smrnas/ | NA |
|  | MaizeSBS | http://mpss.udel.edu/maize/ | 6530566 |
|  | GSE15286 | NCBI GEO | 4183200 |
|  | CSPSR | http://smallrna.udel.edu/index.php | 4216274 |
| *S．lycopersicum* | GSE12081 | NCBI GEO | 275404 |
|  | CSPSR | http://smallrna.udel.edu/index.php | 4957245 |
| *P．patens* | GSE5103 | NCBI GEO | 187034 |
|  | GSE12468 | NCBI GEO | 1037314 |
| *S．moellendorffii* | GSE7320 | NCBI GEO | NA |
| *P．trichocarpa* | Ptr_sRNA | doi:10.1186/1471-2164-8-481 | 13516 |
|  | CSPSR | http://smallrna.udel.edu/index.php | 4334751 |
| *C. sinensis* | GSE18207 | NCBI GEO | 5687010 |
|  | CSPSR | http://smallrna.udel.edu/index.php | 2793114 |
| *G.max* | GSE21825 | NCBI GEO | 1709491 |
| *P. concorta* | MyRNA | ftp://ftp03.bcgsc.ca/public/pine_rice_smRNA/ | NA |
| *T.aestivum* | CSPSR | http://smallrna.udel.edu/index.php | 3151821 |
| *H.vulgare* | CSPSR | http://smallrna.udel.edu/index.php | 3165390 |
| *S.bicolor* | CSPSR | http://smallrna.udel.edu/index.php | 4068164 |
| *P.virgatum* | CSPSR | http://smallrna.udel.edu/index.php | 2978151 |
| *L. sativa* | CSPSR | http://smallrna.udel.edu/index.php | 1797130 |
| *N. tabacum* | CSPSR | http://smallrna.udel.edu/index.php | 2907544 |
| *V. vinifera* | CSPSR | http://smallrna.udel.edu/index.php | 2759176 |
|  | GSE18405 | NCBI GEO | 4272480 |
| *C. rumphii* | CSPSR | http://smallrna.udel.edu/index.php | 3785351 |
| *M．truncatula* | GSE13761 | NCBI GEO | 1974436 |
|  | GSE15438 | NCBI GEO | 81949 |
| ^a^ Name or accession number of the databases  ^b^ Also include Digital Object Identifier (DOI) for journal papers. NCBI GEO: http://www.ncbi.nlm.nih.gov/gds  ^c^ The mean of the total reads of samples in a given database, to which the abundances of small RNAs were normalized. NA, not applicable because only one sample in the database. | | | |
